# Density-gradient centrifugation enables the purification of cultured corneal endothelial cells for cell therapy by eliminating senescent cells

**DOI:** 10.1038/srep15005

**Published:** 2015-10-07

**Authors:** Naoki Okumura, Ayaka Kusakabe, Hiroatsu Hirano, Ryota Inoue, Yugo Okazaki, Shinichiro Nakano, Shigeru Kinoshita, Noriko Koizumi

**Affiliations:** 1 Department of Biomedical Engineering, Faculty of Life and Medical Sciences, Doshisha University, Kyotanabe, Japan; 2 Department of Frontier Medical Science and Technology for Ophthalmology, Kyoto Prefectural University of Medicine, Kyoto, Japan

## Abstract

The corneal endothelium is essential for maintaining corneal transparency; therefore, corneal endothelial dysfunction causes serious vision loss. Tissue engineering-based therapy is potentially a less invasive and more effective therapeutic modality. We recently started a first-in-man clinical trial of cell-based therapy for treating corneal endothelial dysfunction in Japan. However, the senescence of corneal endothelial cells (CECs) during the serial passage culture needed to obtain massive quantities of cells for clinical use is a serious technical obstacle preventing the push of this regenerative therapy to clinical settings. Here, we show evidence from an animal model confirming that senescent cells are less effective in cell therapy. In addition, we propose that density-gradient centrifugation can eliminate the senescent cells and purify high potency CECs for clinical use. This simple technique might be applicable for other types of cells in the settings of regenerative medicine.

The cornea is transparent tissue exposed to the outer environment and serves as the transparent “window” of the eye to allow the entry of light. The corneal endothelium is responsible for maintenance of corneal transparency as a result of regulation by the corneal endothelium pump and barrier function. The proliferative ability of the corneal endothelium is severely limited^1^,^2^; consequently, severe damage to the corneal endothelium due to pathological conditions, such as endothelial corneal dystrophies and surgical trauma, impair corneal transparency and ultimately induce bullous keratopathy with serious vision loss. Corneal transplantation is currently the only therapeutic choice, but a worldwide shortage of donor corneas, the difficulty of the surgical procedure, and graft failure in both acute and chronic phases encourages researchers to develop tissue engineering-based therapies^3^.

A fundamental difficulty for the establishment of a tissue engineering-based therapy is the development of a cell cultivation protocol for clinical application^4^. Many researchers, including us, have devoted their efforts to establishing cell culture protocols^5^,^6^,^7^,^8^,^9^,^10^,^11^. Indeed, we are currently culturing CECs of Good Manufacturing Practice (GMP) grade in the cell-processing center for clinical applications^4^, and have successfully treated the patients with those cells (not published). However, an unresolved problem is the occurrence of cellular senescence, where the cells exhibit morphological changes such as cell enlargement, vacuolization, and multinucleus formation^12^,^13^, during serial passage culture aimed at generating massive numbers of cells for clinical use.

Here, we provide evidence to show that senescent phenotype CECs were less effective in cell-based therapy in an animal model and that non-senescent phenotype cells should be used clinically. We also proposed a simple procedure for purification of cultured human CECs (HCECs) by eliminating the senescent HCECs by density-gradient centrifugation.

## Results

### Senescent CECs *in vitro* and *in vivo*

**Figure 1:**
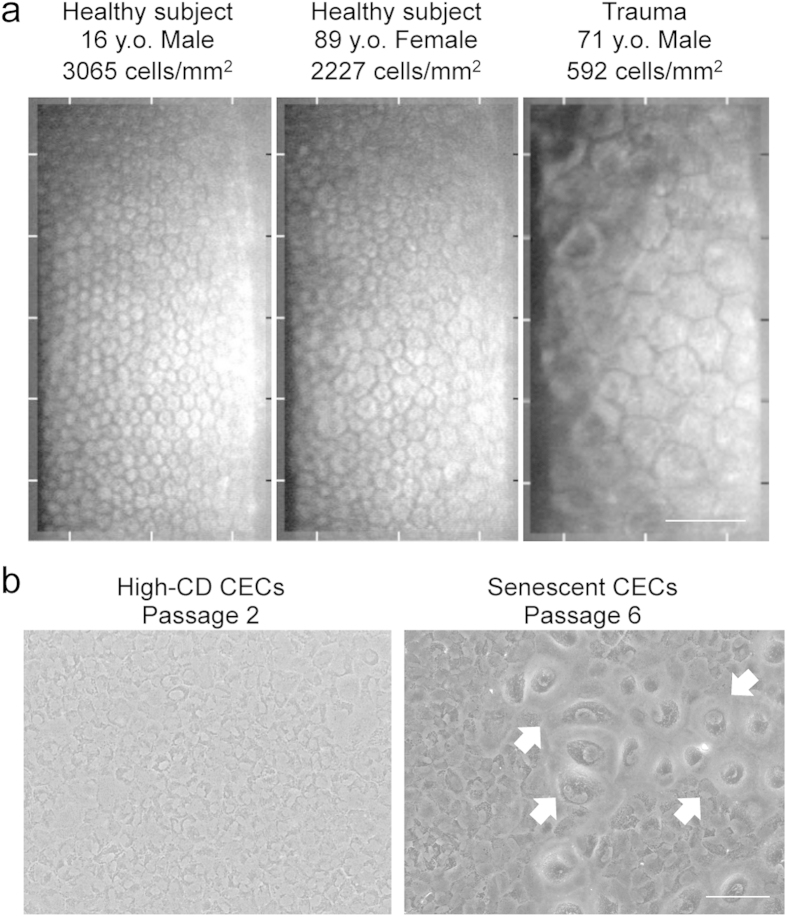
Cellular senescence of CECs *in vivo* and *in vitro*. (**a**) Representative corneal endothelium images obtained by non-contact specular microscopy are shown. Left: A 16-year-old healthy young subject, middle: An 89-year-old healthy elderly subject with relatively low cell density (CD) due to aging, and right: A 71-year-old with low CD CECs due to corneal trauma. Scale bar: 100 μm. (**b**) HCECs were cultured from a human donor cornea and passaged for expansion culture. Left: representative phase contrast images of HCECs passaged 1 time after primary culture with high CD cells. Right: representative phase contrast images of HCECs passaged 6 times; senescent cells are visible within the cultured cell population. Arrows indicate senescent cells. Scale bar: 100 μm.

The cell density of the corneal endothelium is approximately 3000 cells/mm^2^ at the age of 10–20 years, then it gradually decreases with aging at 0.3–0.6% per year, and pathological conditions induce the rapid loss of cell density^14^,^15^. Representative corneal endothelium images obtained by non-contact specular microscope showed that the cell density (CD) is lower in 89-year-old healthy elderly subjects than in 16-year-old healthy young subjects due to aging. Corneal trauma also induced the remaining cells to enlarge and flatten, associated with the cell density decrease (Fig. 1a). We successfully cultured HCECs that maintained a high CD and showed less variation in morphology (Fig. 1b; left). However, the numbers of low CD-HCECs with morphological senescence features, such as enlargement, flattening, vacuolization, and multiple nuclei, sometimes spontaneously increase after passaging the culture 5–8 times (Fig. 1b; right).

### Effect of cell density on cell therapy

**Figure 2:**
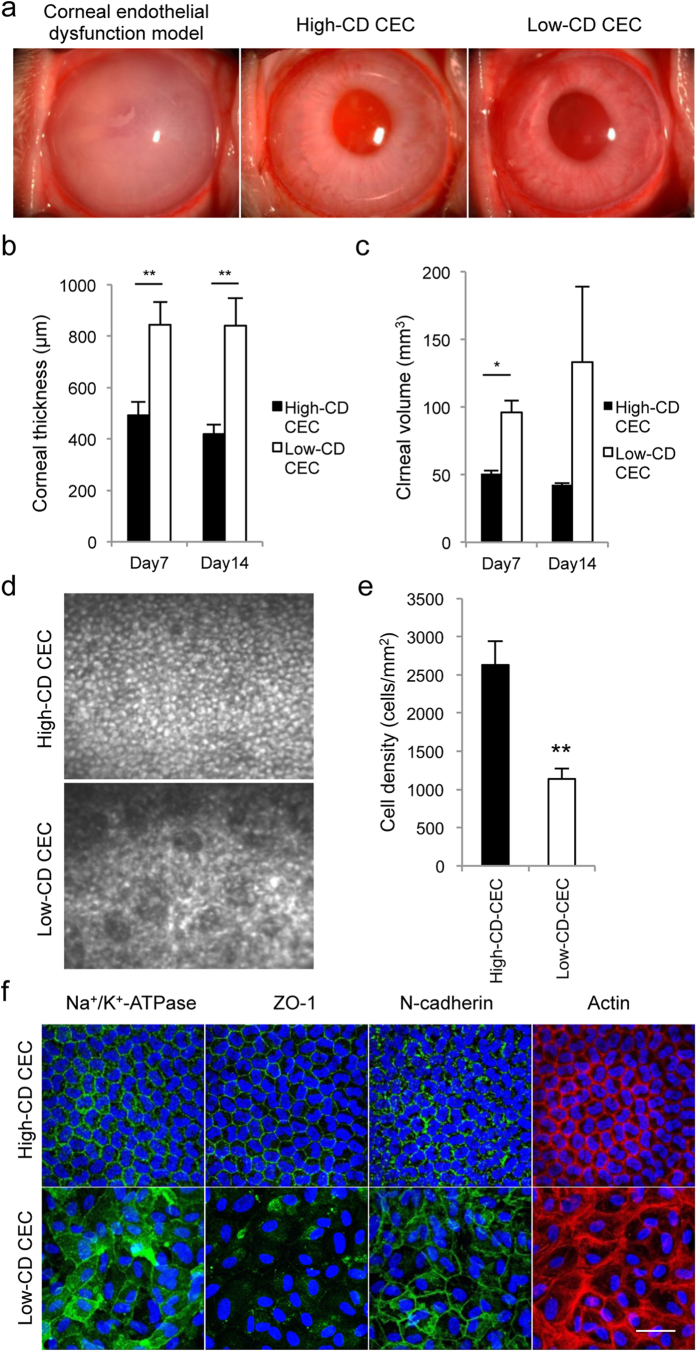
Effect of cellular senescence on cell-based therapy in the corneal endothelial dysfunction rabbit model. (**a**) The corneal endothelial dysfunction model was created by mechanically removing the rabbit corneal endothelium. A total of 5.0 × 10^5^ high-CD or low-CD RCECs was injected, together with ROCK inhibitor, into the anterior chamber, followed by maintenance in a face down position for 3 hours (n = 6). The corneal endothelial dysfunction model in which RCECs were not injected was used as a control (n = 3). Representative slit lamp photograph images are shown. Corneal transparency was restored in endothelial dysfunction models by intracameral injection of high CD RCECs with ROCK inhibitor, while the controls exhibited hazy corneas due to corneal endothelial dysfunction. Interestingly, senescent RCECs with low CD were also able to restore corneal transparency. (**b**, **c**) The mean central corneal thickness and corneal volume evaluated by Pentacam^®^ at 7 and 14 days after cell injection are shown as a graph. The corneal thickness and corneal volume were significantly reduced in the eyes injected with high CD RCECs when compared to eyes injected with low-CD CECs. *P < 0.01, **P < 0.05. (**d**) Regenerated corneal endothelium following injection of high-CD and low-CD RCECs was evaluated by contact specular microscopy at 14 days. (**e**) The mean cell density of regenerated corneal endothelium was analyzed. The CD of the regenerated corneal endothelium was significantly higher in the eyes injected with high CD-CECs than with low-CD senescent CECs. **P < 0.05. (**f**) Function-related markers of CECs (Na^+^/K^+^-ATPase, ZO-1, and N cadherin) were immunostained in the regenerated corneal endothelium. Phalloidin staining was also performed to evaluate the actin cytoskeleton. Na^+^/K^+^-ATPase, ZO-1, and N-cadherin were expressed in all regenerated CECs in eyes injected with high-CD CECs, while expression of these markers was partially disrupted in the CECs in eyes injected with low-CD CECs. Actin was distributed in the cell cortex in the eyes injected with high-CD CECs, while cortical actin distribution showed irregularity and the presence of stress fibers in the eyes injected with low-CD CECs. Nuclei were stained with DAPI. Scale bar: 100 μm.

We were motivated to evaluate the effect of cell senescence on cell-based therapy and conducted experiments using a rabbit corneal endothelial dysfunction model. In accordance with our previous report^16^, corneal transparency was restored in endothelial dysfunction models by intracameral injection of high CD rabbit CECs (RCECs) with ROCK inhibitor, while the controls exhibited hazy corneas due to corneal endothelial dysfunction. Interestingly, senescent RCECs with low CD were able to restore corneal transparency similar to high-CD RCECs (Fig. 2a). However, the corneal thickness and corneal volume, which are indexes of corneal endothelial function, were significantly reduced in the eyes injected with high CD RCECs when compared to eyes injected with low-CD CECs (Fig. 2b,c). The corneal endothelium was regenerated following injection of either high- or low-CD CECs, but the CD of regenerated corneal endothelium was significantly higher in the eyes injected with high CD-CECs than with low-CD senescent CECs (2630.0 cells/mm^2^ and 1137.0 cells/mm^2^, respectively) (Fig. 2d,e). In accordance with these clinical indicators, fluorescent staining demonstrated that the function-related markers Na^+^/K^+^-ATPase (pump function), ZO-1 (tight junction), and N-cadherin (adherent junction) were expressed in all regenerated CECs in eyes injected with high-CD CECs, while expression of these markers was partially disrupted in the CECs in eyes injected with low-CD CECs. Actin was distributed in the cell cortex similar to its distribution in healthy cells in the eyes injected with high-CD CECs, while cortical actin distribution showed irregularity, with stress fibers, in the eyes injected with low-CD CECs, suggesting that the functional and morphological recovery is poor when elicited by senescent cells (Fig. 2f).

### Phenotypic analysis of senescent cells

**Figure 3:**
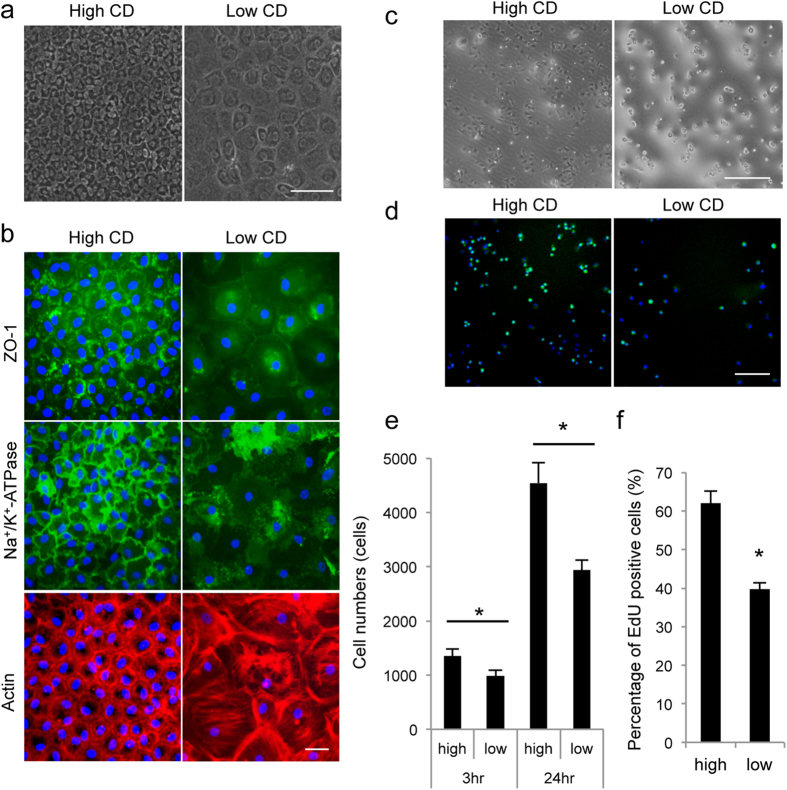
Phenotypic analysis of low-CD RCECs. (**a**) Representative phase contrast images of high-CD and low-CD RCECs are shown. RCECs with a CD of 2540.4 ± 64.9 cells/mm^2^ were used as high-CD cell and 720.2 ± 29.4 cells/mm^2^ were used as low-CD cells for this study. Scale bar: 100 μm. (**b**) ZO-1, Na^+^/K^+^-ATPase and actin were stained in high-CD and low-CD RCECs. Nuclei were stained with DAPI. Expressions of ZO-1 and of Na^+^/K^+^-ATPase were partially disrupted in low-CD CECs, but these expressions occurred in all high-CD CECs. Disruption of actin at the cell cortex was also observed, along with the formation of stress fibers, but only in low-CD CECs. Scale bar: 50 μm. (**c**) High-CD and low-CD RCECs were seeded at a density of 5 × 10^3^ cells/cm^2^ and cultured for 24 hours. Scale bar: 50 μm. (**d**) The effect of CD on cell proliferation was evaluated using 5-ethynyl-2′-deoxyuridine (EdU) Click-iT^®^ imaging kits. (**e**) The number of attached cells was calculated using the CellTiter-Glo^®^ Luminescent Cell Viability Assay at 3 and 24 hours after seeding (n = 6). The numbers of adhered cells were significantly lower in low-CD CECs than in high-CD CECs. *P < 0.01. (**f**) EdU positive cells were counted after 9 hours of incubation (n = 3). The numbers of EdU positive cells were significantly lower in low-CD CECs than in high-CD CECs. *P < 0.01. Experiments were performed in duplicate.

Phenotypic analysis of senescent cells revealed enlargement and flattening in cells with low CD. RCECs underwent senescence spontaneously after 4–5 passages but formed monolayer sheet structures with cobblestone-like morphology and were used for the experiment (Fig. 3a). Expressions of ZO-1, which represents the barrier function, and of Na^+^/K^+^-ATPase, which represents pump function, were are partially disrupted in low-CD CECs, while they were expressed in all high-CD CECs. Disruption of actin at the cell cortex was also observed, with the formation of stress fibers, but only in low-CD CECs (Fig. 3b). Cell adhesion and proliferation potencies were significantly decreased in low-CD CECs when compared to high-CD CECs (Fig. 3c–f).

### RCEC purification by density-gradient centrifugation

**Figure 4:**
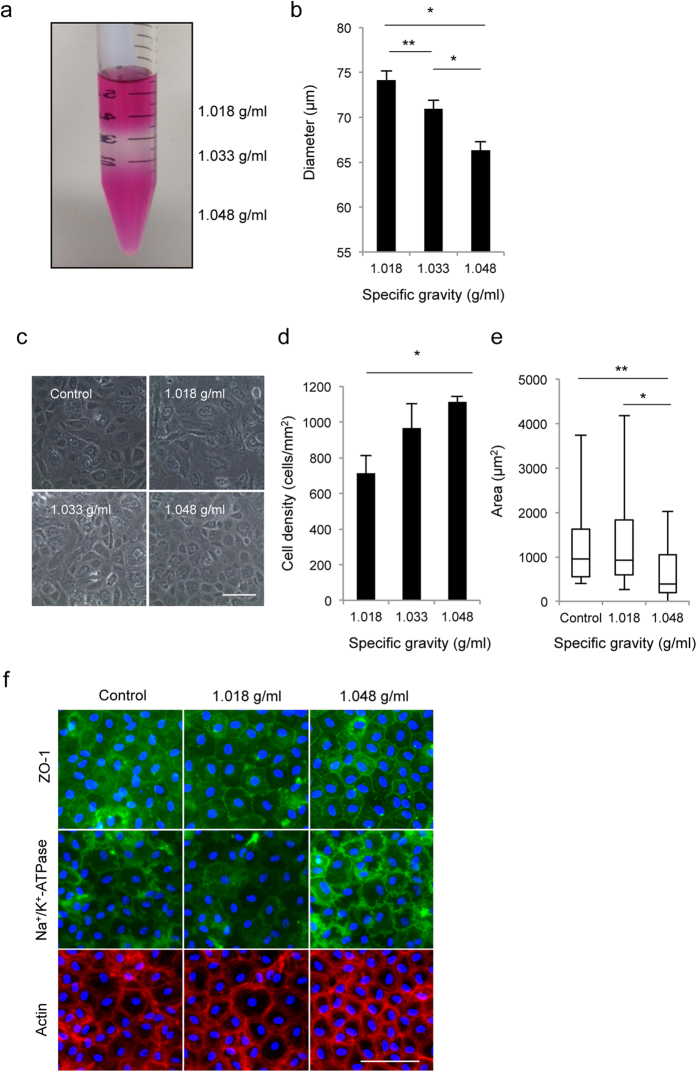
Density-gradient centrifugation of RCECs. (**a**) The cultured RCECs, including senescent RCECs with low-CD RCECs, were centrifuged through OptiPrep^TM^ Density Gradient Medium at 800 g for 15 minutes. (**b**) The diameter of cells recovered from density gradient media with different specific gravity was evaluated by flow cytometry. RCECs were separated according to cell size. *P < 0.01, **P < 0.05. Experiments were performed in triplicate. (**c**) Cells recovered from density gradient media of different specific gravity were cultured for 2 weeks after cells reached confluence. Representative phase contrast images are shown. (**d**,**e**) Cell density and cell area were determined with Image J^®^ (NIH) software. The CD was significantly higher in the CECs recovered from the 1.048 g/ml fraction than from the 1.018 and 1.033 g/ml fractions. The variation in cell size was smaller in the 1.048 g/ml fraction cells than in the control cells. *P < 0.01, **P < 0.05. Experiments were performed in triplicate. (**f**) Representative images of ZO-1, Na^+^/K^+^-ATPase, and actin staining are shown. Nuclei were stained with DAPI. Expressions of ZO-1 and Na^+^/K^+^-ATPase were partially disrupted in the control, whereas RCECs recovered from the 1.048 g/ml fraction fully expressed these function-related proteins. Scale bar: 100 μm.

Next, we hypothesized that senescent cells can be separated by density-gradient centrifugation according their specific gravity because the senescent CECs are enlarged and flattened. Additionally, the cell size is believed to be the most valid indicator for corneal endothelial healthiness in clinical settings, which gave us reason to eliminate large cells as senescent cells. We tried to separate RCECs from a mixture of high-CD CECs and spontaneously-appearing senescent CECs into several fractions according to their specific gravity using OptiPrep^TM^ Density Gradient Medium (Fig. 4a). Flow cytometry showed that CECs were separated according to cell size; i.e., the heavier specific gravity fraction included smaller diameter CECs and the lighter specific gravity fraction included the larger CECs (Fig. 4b). After seeding those cells, the CD was significantly higher in the CECs recovered from the 1.048 g/ml fraction than from the 1.018 and 1.033 g/ml fractions (Fig. 4c,d). In agreement with the smaller cell diameter of CECs recovered from the 1.048 g/ml fraction, the variation in cell size was smaller than those from 1.018 and 1.033 g/ml fractions after reaching confluence in culture. Additionally, the variation in cell size was smaller in the 1.048 g/ml fraction cells than in the control cells (Fig. 4e). Expressions of ZO-1 and Na^+^/K^+^-ATPase were partially disrupted in the control, whereas all cells recovered from the 1.048 g/ml fraction expressed these function-related proteins. Actin staining showed that cells recovered from 1.048 g/ml fraction were less variable in size and maintained their hexagonality (Fig. 4f).

### HCEC purification by density-gradient centrifugation

**Figure 5:**
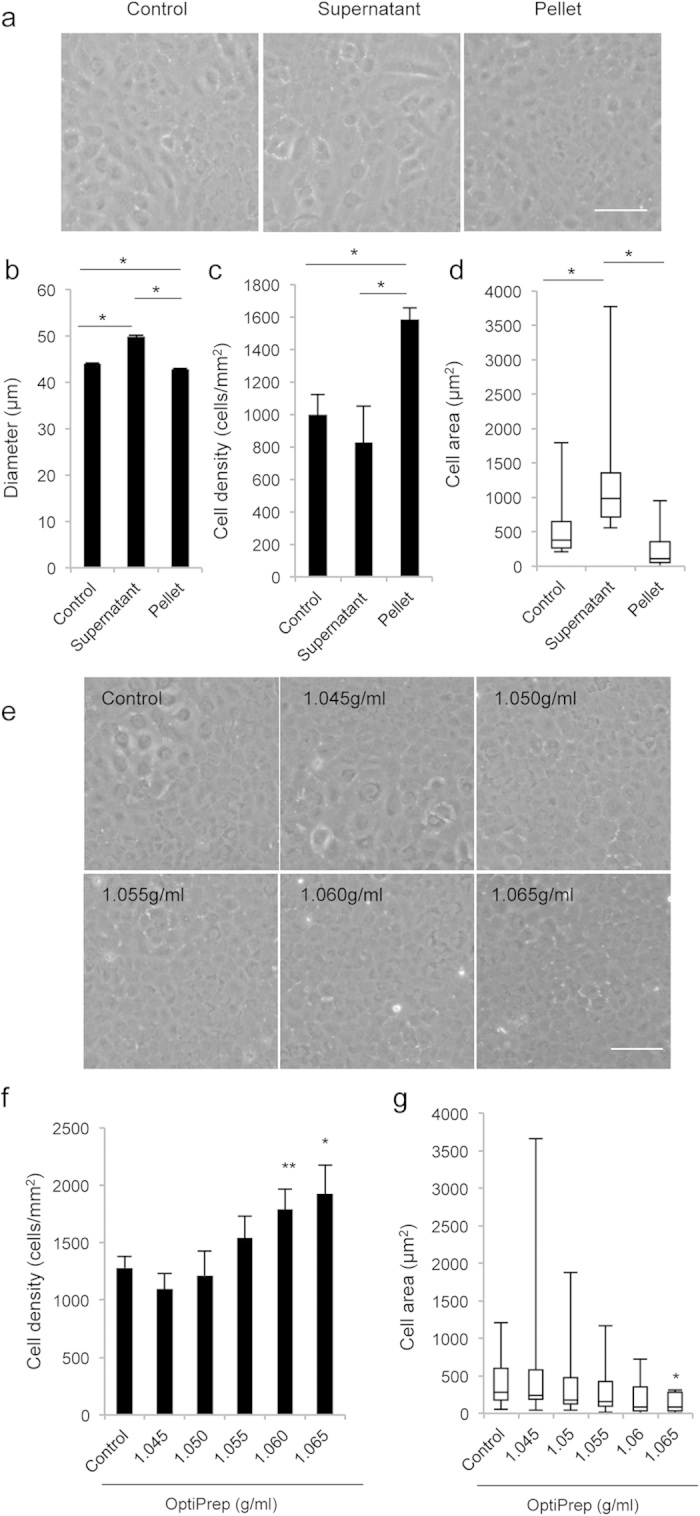
Density-gradient centrifugation of HCECs. (**a**) The cultured HCECs, including senescent cells with a low CD, were centrifuged through 1.060 g/ml density gradient medium at 800 g for 15 minutes. The cells recovered from supernatant and pellet were then seeded at the same cell numbers and cultured for 2 weeks after cells reached confluence. (**b**) The diameter of cells recovered from the supernatant and pellet were evaluated by flow cytometry. The mean diameter of the pellet-derived cells was 42.9 μm, while that of supernatant-derived cells was 49.9 μm. *P < 0.01. Experiments were performed in triplicate. (**c,d**) Cell density (CD) and cell area were determined with Image J^®^ (NIH) software. The CD of pellet-derived HCECs was 1584.5 cells/mm^2^, while that of supernatant-derived HCECs was 827.8 cells/mm^2^. *P < 0.01. Experiments were performed in triplicate. (**e**) HCECs were centrifuged through 1.045, 1.050, 1.055, 1.060, and 1.065 g/ml density gradient medium at 800 g for 15 minutes. HCECs recovered from the pellet were seeded at the same cell numbers and cultured for 2 weeks after the cells reached confluence. (**f**,**g**) Cell density and cell area were determined with Image J^®^ (NIH) software. The 1.065 g/ml density gradient medium enabled purification of HCECs with higher CD and less size variation. *P < 0.01, **P < 0.05.

We conducted experiments to evaluate whether cultured HCECs can be purified by separating out senescent cells by density-gradient centrifugation. HCECs recovered from the supernatant and from the precipitated pellet after centrifugation in 1.060 g/ml density gradient medium were subsequently cultured (Fig. 5a). Flow cytometry demonstrated a mean diameter of pellet-derived cells of 42.9 μm, while that of supernatant-derived cells was 49.9 μm (Fig. 5b). Culture of the separated cells gradient revealed that pellet-derived HCECs formed monolayer cell sheets with a hexagonal cobblestone-like phenotype with a CD of 1584.5 cells/mm^2^ while the supernatant-derived HCECs had an elongated phenotype with a CD of 827.8 cells/mm^2^ (Fig. 5c). The cell area was significantly smaller for the pellet-derived HCECs than for the supernatant-derived HCECs (Fig. 5d).

We also conducted experiments to optimize the specific gravity of the density gradient medium for removal of senescent cells from the HCECs for clinical application. Of note, HCECs recovered from the pellet following centrifugation in the density gradient medium could be cultured with a normal cell passage procedure (Fig. 5e). The 1.065 g/ml density gradient medium enabled purification of HCECs with a cell density of 1924.3 cells/mm^2^ (Fig. 5f). Variation in the cell sizes of HCECs cultured from the pellet was smaller than in the control, suggesting that density gradient centrifugation minimizes the variation in cell size (Fig. 5g).

## Discussion

Regenerative medicine for treating corneal endothelial dysfunction has been anticipated to provide an alternative therapeutic choice to corneal transplantation^3^. Cultured sheet transplantation and cell-based therapy have been researched in animal models^16^,^17^,^18^,^19^,^20^,^21^. For instance, we showed that corneal endothelium can be regenerated in rabbit and monkey corneal endothelial dysfunction models by injecting a cultured CEC suspension combined with a Rho-associated kinase (ROCK) inhibitor^16^. In addition, we recently started a first-in-man clinical trial at Kyoto Prefectural University of Medicine after obtaining the necessary approval (Clinical trial registration: UMIN000012534)^4^. In this clinical trial, we isolated and cultured CECs from human donor corneas and injected them, together with ROCK inhibitor, into the anterior chamber of the patients (unpublished data).

No protocol specifically designed for clinical application can yet overcome the problems of the limited proliferative ability of CECs and their tendency to undergo fibroblastic transformation^4^. Consequently, our research group and others have been continuously striving towards the development of a successful culture method^5^,^6^,^9^,^10^,^11^,^22^,^23^. For instance, we reported that ROCK inhibitor^5^ and conditioned medium obtained from GMP-grade human bone marrow-derived mesenchymal stem cells (BM-MSCs)^8^ enhanced CEC proliferation. We also showed that the fibroblastic transformation of CECs is caused by activation of transforming growth factor beta (TGF-β) signaling, and that inhibition of TGF-β signaling by small molecules enables CECs to counteract fibroblastic changes and maintain the endothelial phenotype^9^. One unresolved clinical issue is that several passages are required to obtain large numbers of cells (e.g.; 5 passages enables the treatment of 100–200 patients from 1 donor cornea), but serial passaging also leads to a senescent cell phenotype.

Morphological features of senescent cells usually include cell enlargement, flattening, vacuolization, and occasionally multinucleation^12^. Here, we demonstrated that senescent CECs can be separated by differences in specific gravity. Possible explanations include: 1) the lower nucleus/cytoplasm ratio of senescent CECs yields a lower specific density, as the larger volume of cytoplasm has a less dense internal structure than the nucleus; and 2) vacuolization of senescent CECs results in a lower specific gravity, as the vacuoles have a lower density than the rest of the cytoplasm. We also showed that low-CD CECs exhibited these senescence features, together with decreases in the expression of function-related proteins, adhesion potency, and proliferative ability. Cellular senescence can be induced by a variety of triggers in various cell types, and no definitive marker has been established; therefore, a set of criteria is required for each tissue^12^,^13^.

One potential biomarker for senescence of corneal endothelium is SA-β-gal, an indicator of senescent cells, as it is expressed at higher levels in the corneal endothelium of elderly individuals than of young individual^24^. The expression of p21; the main driving force for the induction of the senescence program, and p16, a p21-independent senescence-determining molecule, are expressed at higher levels in older individuals than in younger individuals^1^,^2^. The evidence that low-CD CECs are senescent cells, as determined by an established set of criteria for corneal endothelial senescence, needs further investigation, but we propose that CD is a simple and practical criterion that can predict the outcome of corneal endothelial cell-based therapy.

Magnetic affinity cell separation (MACS)^25^ and fluorescence-activated cell sorting (FACS)^26^ were reported to purify cultured HCECs. However, these methods are not ideal for therapeutic application, because of drawbacks that include the use of antibodies, the cost issues to establish the GMP-grade MACS or FACS, and the fibroblastic transformation after cell dissociation for cell sorting. In the current study, we demonstrated that density-gradient centrifugation could purify CECs by eliminating the senescent cells based on cell size. Senescent cells exhibit a senescence-associated secretory phenotype (SASP), and the SASP components can trigger senescence in neighboring cells in a paracrine manner^12^,^27^. This simple technique of density-gradient centrifugation is applicable for final cell preparation for the patient but can also be used for each passage procedure to eliminate senescent cells throughout the cultivation period. In conclusion, density-gradient centrifugation, by separating out senescent cells, will enable the purification of HCECs for cell based therapy for treating corneal endothelial dysfunction. This technique might also be applicable for other types of cells in the settings of regenerative medicine.

## Methods

### Ethics statement

Animals were housed and treated in accordance with the ARVO Statement for the Use of Animals in Ophthalmic and Vision Research. The rabbit experiments were performed at Doshisha University (Kyoto, Japan) according to the protocol approved by that university’s Animal Care and Use Committee (Approval No. A15011-2). Human donor corneas were obtained from SightLife^TM^ (http://www.sightlife.org/, Seattle, WA) for research purposes.

### Cell Culture

Thirty rabbit eyes were used for the rabbit CEC (RCEC) culture. The RCECs were cultivated as described previously^16^. Briefly, Descemet’s membrane with RCECs was stripped and incubated in 0.6 U/mL of Dispase II (Roche Applied Science, Penzberg, Germany) to release the RCECs. The isolated RCECs were resuspended in culture medium and plated in 1 well of a 6-well plate coated with cell attachment reagent (FNC Coating Mix^®^, Athena Environmental Sciences, Inc., Baltimore, MD). All primary cell cultures and serial passages of RCECs were performed in growth medium composed of Dulbecco’s modified Eagle’s medium (Life Technologies Corp., Carlsbad, CA) supplemented with 10% fetal bovine serum (FBS), 50 U/mL penicillin, 50 μg/mL streptomycin, and 2 ng/mL fibroblast growth factor 2 (Life Technologies Corp.). Cultivated RCECs at passages 1 through 3 were used for all experiments. RCECs with a CD of 2540.4 ± 64.9 cells/mm^2^ were used as high-CD cells and those with a CD of 720.2 ± 29.4 cells/mm^2^ were used as low-CD cells for this study.

A total of six human donor corneas (from persons >40 years of age) were used for cultivation of human CECs (HCECs) by the protocol described previously. Briefly, Descemet’s membranes containing the HCECs were stripped from donor corneas and the membranes were digested with 1 mg/mL collagenase A (Roche Applied Science) at 37 °C for 12 h. The HCECs were seeded in one well of a 48-well plate. Plates had previously been coated with laminin E8 fragments (iMatrix-511; Nippi, Incorporated, Tokyo, Japan) (2.0 μg/cm^2^)^11^. The culture medium was prepared according to published protocols^8^.

First, BM-MSCs were cultured according to previously reported protocols. Briefly, BM-MSCs were plated at a density of 1.3 × 10^4^ cells/cm^2^ and cultured for 24 hours in DMEM supplemented with 10% FBS, 100 U/mL penicillin, and 100 μg/mL streptomycin. Then, basal medium for HCECs was prepared (OptiMEM-I (Life Technologies Corp.) containing 8% FBS, 5 ng/mL epidermal growth factor (Sigma-Aldrich Co., St. Louis, MO), 20 μg/mL ascorbic acid (Sigma-Aldrich Co.), 200 mg/L calcium chloride, 0.08% chondroitin sulfate (Wako Pure Chemical Industries, Ltd., Osaka, Japan), 50 μg/mL gentamicin, and 10 μM SB431542 (Merck Millipore, Billerica, MA)) and conditioned by culturing BM-MSCs for 24 hours. Finally, the basal medium conditioned with BM-MSCs was collected for use as the culture medium for HCECs.

### Injection of RCECs into a corneal endothelial dysfunction model

The rabbit corneal endothelial dysfunction models were created following lens removal by mechanically scraping the corneal endothelium from the Descemet’s membrane with a 20-gauge silicone needle (Soft Tapered Needle; Inami & Co., Ltd., Tokyo, Japan) as described previously^16^. A total of 5.0 × 10^5^ RCECs, suspended in 200 μl of DMEM supplemented with 100 μM of Y-27632 (Wako Pure Chemical Industries, Ltd.), was injected into the anterior chamber of the corneal endothelial dysfunction model and the eyes were kept in the face-down position for 3 hours under general anesthesia. Anterior segments and corneal endothelium were evaluated by slit-lamp microscopy and contact specular microscopy (Konan scanning slit specular microscope, Konan Medical, Nishinomiya, Japan) for 2 weeks. Corneal thickness and corneal volume were evaluated with a Pentacam^®^ (OCULUS Optikgeräte GmbH, Wetzlar, Germany).

### Fluorescence staining

Rabbit corneal specimens were fixed in 4% formaldehyde and incubated for 30 minutes in 1% bovine serum albumin (BSA) to block nonspecific binding. Corneas were examined by actin staining performed with a 1:400 dilution of Alexa Fluor^®^ 546 conjugated Phalloidin (Life Technologies Corp.). For immunohistochemical analyses, specimens were incubated with primary antibodies against Na^+^/K^+^-ATPase (1:300, Upstate Biotechnology, Lake Placid, NY), ZO-1 (1:300, Life Technologies Corp.), and N-cadherin (1:300, BD Biosciences, San Jose, CA), and then Alexa Fluor^®^ 488-conjugated goat anti-mouse (Life Technologies Corp.) was used as a secondary antibody at a 1:1000 dilution. Proliferative cells were evaluated by 5-ethynyl-2 Click-iT^®^ EdU imaging kits (Life Technologies Corp.) according to the manufacturer’s instructions. Briefly, the RCECs (1 × 10^4^ cells/well) were cultured in a 96-well plate and incubated with 10 μM EdU for 6 h at 37 °C. Following fixation with 4% paraformaldehyde and permeabilization with 0.3% Triton^**®**^X-100 (Nacalai Tesque, Kyoto, Japan), the RCECs were incubated with a reaction cocktail for 30 min at room temperature. Nuclei were stained with DAPI (Vector Laboratories, Burlingame, CA). The slides were examined with a fluorescence microscope (TCS SP2 AOBS; Leica Microsystems, Wetzlar, Germany).

### Density-gradient centrifugation

The cultured RCECs or HCECs were suspended in DMEM or Opti-MEM^®^ Reduced Serum Medium, respectively. The OptiPrep^TM^ Density Gradient Medium (Sigma-Aldrich Co.) was then added and the cells were centrifuged at 800 g for 15 minutes. RCECs or HCECs were recovered from the pellet or supernatant, respectively.

### Flow cytometry

RCECs or HCECs recovered following density gradient centrifugation were suspended in DMEM or OptiMEM-I. Cells were evaluated by flow cytometry using the BD Accuri ^TM^ (BD Biosciences), and the cell diameter was determined using a SPHERO™ Flow Cytometry Size Standard Kit (Spherotech, Inc. Lake Forest, IL).

### Statistical analysis

The statistical significance (P-value) of differences between mean values of the two-sample comparison was determined with the Student’s t-test. The comparison of multiple sample sets was analyzed using Dunnett’s multiple-comparison test. The values shown in the graphs represent the mean ± SEM.

## Additional Information

**How to cite this article**: Okumura, N. *et al*. Density-gradient centrifugation enables the purification of cultured corneal endothelial cells for cell therapy by eliminating senescent cells. *Sci. Rep*. **5**, 15005; doi: 10.1038/srep15005 (2015).
